# Comparative analysis of animal lifespan

**DOI:** 10.1007/s11357-023-00984-2

**Published:** 2023-10-27

**Authors:** Nicole C. Riddle, Peggy R. Biga, Anne M. Bronikowski, James R. Walters, Gerald S. Wilkinson, IISAGE Consortium, Jingyue Ellie Duan, Anthony Gamble, Erica Larschan, Richard P. Meisel, Ritambhara Singh, Ashley Webb

**Affiliations:** 1 Department of Biology, University of Alabama at Birmingham, Birmingham, AL USA; 2 Department of Integrative Biology, Kellogg Biological Station, Michigan State University, Hickory Corners, MI USA; 3 Department of Ecology and Evolutionary Biology, The University of Kansas, Lawrence, KS USA; 4 Department of Biology, University of Maryland, College Park, MD USA

**Keywords:** Comparative biology, Aging, Models of aging, Lifespan

## Abstract

Comparative studies of aging are a promising approach to identifying general properties of and processes leading to aging. While to date, many comparative studies of aging in animals have focused on relatively narrow species groups, methodological innovations now allow for studies that include evolutionary distant species. However, comparative studies of aging across a wide range of species that have distinct life histories introduce additional challenges in experimental design. Here, we discuss these challenges, highlight the most pressing problems that need to be solved, and provide suggestions based on current approaches to successfully carry out comparative aging studies across the animal kingdom.

Comparative analyses of aging and lifespan have great potential to lead to new insights into both the species-specific and general properties of aging [1–3]. Due to the immense societal and financial cost of health issues that are associated with human aging [4, 5], there is a need to better understand the aging process in general. The traditional model systems of mice, rats, fruit flies, and nematodes have demonstrated that there are many commonalities in how different animal species experience aging, but that there are also important differences [6]. These differences in the aging process are highlighted by studies that look beyond the traditional model systems, as they demonstrate that one can find exceptions to almost any “rule” of aging that has been identified based on the human experience. For example, in contrast to humans, hair graying in chimpanzees (*Pan troglodytes*) increases initially up to midlife but then shows no consistent relationship with age [7]. In some birds such as the common tern (*Sterna hirundo*) and also in reptiles, reproductive capacity does not decrease with age, in contrast to what is seen in humans [8, 9]. These examples illustrate the promise of comparative aging studies of species from across the animal kingdom to gain a better understanding of aging mechanisms.

While comparative aging studies have great promise, they also introduce complexities not encountered when studying a single species. First and foremost, there is a profound need for a methodology that allows researchers to compare lifespans of organisms that might differ by orders of magnitude or have very different life histories. Mayflies (*Ephemera danica*) for example, have an adult lifespan of just 1–2 days [10], while queen termites might live for more than 20 years (e.g. fungus-growing termite *Macrotermes bellicosus* [11]; for a review, see [12]), and periodic cicadas (*Magicicada ssp*) spend 17 years underground as nymphs before emerging [13]. Similarly, short-lived mammals might live only a few months (Giant Sunda Rat, *Sundamys infraluteus*) while long-lived species survive dozens of years [14]. Among sharks and mussels, lifespans in excess of 400 years have been reported [15, 16]. With this immense diversity in absolute lifespan comes a range of different life histories that can include different portions of the time before sexual maturity is reached, metamorphosis, various types of dormancies, and much more. Given this array of differences, a strategy for how to compare lifespan that accounts for this variability is needed.

Currently, there are few approaches to compare the lifespan of diverse species (for an approach for rabbits and humans, see [17]), and most comparative aging studies focus on related species groups (e.g. [18, 19]). As studies are expanded to include more distantly related species with different life history patterns, applying the methods typically used to compare lifespan results in challenges that need to be overcome. Here, we discuss the challenges encountered in comparative aging studies of distantly related animal species and highlight the problems that need to be solved to successfully carry out comparative aging studies across the animal kingdom.

## Measuring lifespan and age

One prerequisite for effective comparative studies of aging is an understanding of how lifespan is measured. Intuitively, this question would seem rather trivial, but even for humans, there are cultural differences in how age is counted. For example, many Asian cultures start the count at birth with 1 to account for the duration of the pregnancy, while most Western cultures start with a count of 0 at birth. Not surprisingly, there is significant variation among research communities in how they measure age for their study species [20–22]. Birth or hatching of the new individual are commonly used markers for many organisms, but others are used as well. Events that start the age clock can include zygote formation, egg laying, or the attainment of certain developmental milestones such as sexual maturity or eye-opening. Given these differences in approaches to how to count an animal’s age, a clear understanding of what ages reported in the literature mean is required for any comparative study, and adjustments might need to be made to compensate for the different counting procedures in the various research communities.

To illustrate the complex nature of the problem, here is a sampling of the methods used to record the age of various study animals that our author team has encountered in their work:

1. *Drosophila melanogaster* (fruit fly): Lifespan in *Drosophila* typically is measured in days from the time an adult fly emerges from the pupae [20]. At this point, the animal is not sexually mature, as this developmental milestone will be reached approximately 8 h after emergence (at 25 °C) [23, 24]. Considering only the time after the adult animal emerges from the pupae for lifespan analysis is a common approach used for many holometabolic insects that undergo complete metamorphosis.
2. *Caenorhabditis elegans* (roundworm): Lifespan in *C. elegans* typically is measured as days after a clear developmental time point, but the specific time points used vary and can include “egg deposition, the emergence of the L1 larvae or the fourth larval stage” [25]. However, other criteria are used as well, as a 2009 publication notes, “in nematodes, life span is typically defined as the number of days an animal remains responsive to external stimuli” [26]. The *C. elegans* embryo develops for about 9 h before the L1 larvae hatch. Larval development is completed in ~ 38 h (at 22 °C) (L1—12 h, L2—8 h, L3—8 h, and L4—10 h), after which an immature adult emerges that will reach sexual maturity after ~ 8 h [27]. The typical lifespan of *C. elegans* reared on UV-killed bacteria is 18–25 days depending on diet etc. [26, 27]. After hatching, animals can also enter a dauer stage (developmental arrest) for up to 4 months before emerging from this arrest as L4 larvae [28]. Depending on which starting point is used to measure age and lifespan, and if the dauer period is included, different fractions of development are included in the measure.
3. *Mus musculus* (mouse): Lifespan for laboratory mice typically is measured as the time (in hours/days/weeks/months) after birth. The animals will reach sexual maturity approximately 4–7 weeks after birth [29].
4. *Gallus gallus domesticus* (chicken): For chickens, the age of the animals is recorded as “days post-hatching”, or the time after the animal hatches from the egg. Chickens typically hatch approximately 21 days after egg-laying (38 °C incubation temperature), and they reach sexual maturity 16–24 weeks after hatching from the egg [30]. Using birth or hatching as the starting point to measure lifespan is a common approach for mammals and vertebrates.
5. *Danio rerio* (zebrafish): For zebrafish, the age of the animals typically is recorded as “days post-fertilization”, or the time after fertilization of the eggs occurs. The immature animals will hatch from the eggs 3–4 days post-fertilization and reach sexual maturity at approximately 3 months of age (28.5 °C rearing temperature) [31]. Measuring the age of the animals from the time of fertilization is a common approach for species with external fertilization (in other fish species, hatching from the egg or birth is used as a starting point).
6. *Tupaia glis belangeri* (Northern tree shrew): Tree shrews are a model system used to study the visual system. In these studies, the age of the animals often is reported as “days of visual experience”, measuring the time since eye-opening (e.g. [32]). This developmental time point is reached approximately 21 days after birth, and the animals will reach sexual maturity approximately 4 months after birth [33, 34].

This small sample of methods used to record age in various animals illustrates that scientists working with different species record the age of their animals in profoundly different ways. For some, such as zebrafish, the entire time an individual exists, from fertilization to death, is considered when recording its lifespan. In contrast, for many other species, significant amounts of time are not included in the time recorded as an animal’s lifespan, as age is counted from a specific developmental time point such as birth or hatching from the egg. Working within related species groups, the methods for counting an animal’s age tend to be similar, making comparisons of lifespan easier, as the measure being compared is the same. However, as the evolutionary divergence between animal groups under study increases, so does the likelihood of encountering lifespans measured by more than one method, leading to challenges in comparing lifespan and age for diverse species.

**Fig. 1:**
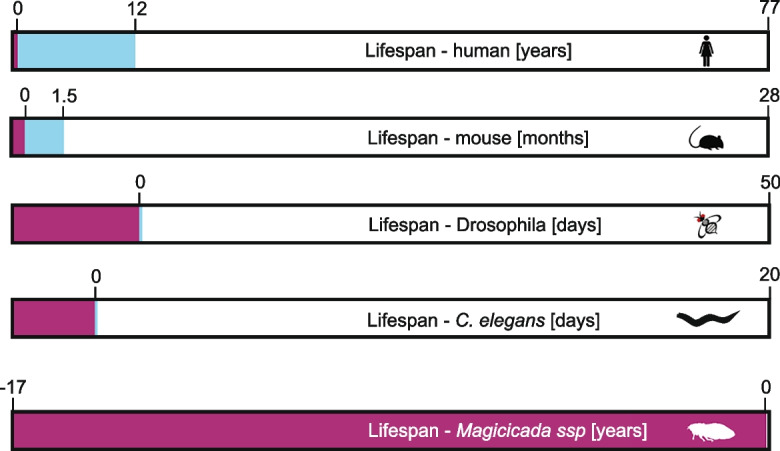
How age is counted differs between organisms. Different scientific communities use different methods for how the age of an organism is counted. This diagram shows the entire life of five organisms from fertilization to death, all scaled to the same length bar. Each bar shows in the red shade the time the organism develops before the “zero” time point when the age count typically starts. The blue shade marks the time before the organism is sexually mature that is included in the age count for each species. For humans (top), the 9 months of development before birth are excluded from the age count, but the approximately 12 years prior to sexual maturity are included in the average 77-year lifespan. Similarly, for mice (second from top), the approximately 20 days of development before birth are excluded from the age count, but the approximately 6 weeks prior to sexual maturity are included in the average 28-month lifespan. For *Drosophila* (middle), the 10 days of development (in the egg and as larva) prior to the eclosion of the adult animal from the pupa are excluded from the age count, while the 8 h prior to sexual maturity are included in the average 50-day lifespan. For *C. elegans* (second from bottom), the 2.5 days prior to the adult stage (2.5 h of development prior to egg laying, 12 h development to hatching, four larval stages) are excluded from the age count, but the 8 h prior to sexual maturity are included in the approximately 20-day lifespan. Finally, in the extreme example of periodic cicadas (bottom), the 4–6 weeks of development in the egg and the 17 years of development underground are excluded from the age count, but the 4–10 days before the adult cicada reaches sexual maturity are in the approximately 1-month lifespan

The biggest difference between the various ways to measure lifespan and age is how “developmental time” is handled. How much of an organism’s lifespan is spent in development to reach the stage relevant for counting age differs significantly between species (Fig. 1). Consider two examples, humans, where lifespan is conventionally measured from birth, and *Drosophila melanogaster*, where lifespan is measured starting at the emergence of the metamorphosed fly from the pupal case. In humans, the 9 months of development in utero before birth are not considered in the lifespan, but the developmental stages prior to reproductive maturity are. Similarly, in *D. melanogaster*, the developmental stages prior to the emergence of the adult fly are not considered when the age of the animal is reported, but as in humans, the time prior to reproductive maturity (~ 8 h) is considered with reported age. However, the lengths of embryogenic development and later developmental stages that are part of the age/lifespan consideration of these two species are very different. Humans live on average 76 years (data for the US; [35]), meaning the 9 months of embryonic/fetal development are about 1% of the total lifespan. In *D. melanogaster*, this fraction is quite different, as the 10 days of embryonic and larval development prior to the eclosion of the adult fly account for about 20% of the average adult lifespan of ~ 50 days [23]. Similarly, the time prior to reaching sexual maturity is quite different in the two species. Humans spend about 16% of their lifespan prior to reaching reproductive maturity at about 12 years of age [36]. In contrast, *D. melanogaster* reach sexual maturity 8 h after eclosion [23], which represents 0.6% of their average lifespan. Thus, in *D. melanogaster*, 20% of the time from fertilization to death is excluded from typical lifespan reports, while in humans, the time from fertilization to birth that is not included in lifespan reports represents only 1% of the total time an animal is alive. This example illustrates how the different methods for recording lifespan for various species groups result in challenges for comparative studies of aging that include species from across the animal kingdom.

In addition to basic differences in how age is counted, there are certain life history events that complicate this issue even further. Diapause is one such life history feature that can complicate lifespan and age estimates in comparative studies. Diapause is a delay or pause during development seen in a variety of species, often due to non-ideal environmental conditions [37, 38]. Diapause can significantly increase the lifespan of an individual, depending on how lifespan is recorded, i.e., if it includes developmental time or not. Diapause occurs in a range of animals, including insects, nematodes, mammals, and fish [39–43]. Diapause can occur at different points in the lifespan. For example, insects can enter diapause as pupa or as adults (e.g. Monarch butterflies) [38]. In mammals, embryonic diapause, also called delayed implantation, occurs in a variety of species [44, 45]. The diapause or “dauer” form of nematodes is well-studied in *C. elegans*, where it can last for up to 4 months (compared to the normal ~ 2-week lifespan) [46], and in *Pristionchus pacificus*, the survival of the dauer larvae has been documented for over 1 year [47]. The short-lived African turquoise killifish *Nothobranchius furzeri (GRZ)* typically spends up to 5–6 months in diapause (can be up to 4–5 years [48]) during embryonic development to avoid unfavorable environmental conditions (lifespan 2–5 months in the wild, up to 12 months in captivity) [49–51]. In this species, the diapause length is known not to affect the lifespan or physiology after diapause exit [52]. However, similar data are not available for most species in which diapause occurs. In addition, for animals that brumate—such as many poikilothermic vertebrates—the duration of this “diapause” can extend over weeks to several years. For most species, it is unknown how diapause and the length of diapause impact the life of the animals afterward and if survivorship curves are different for animals that do and do not experience diapause. Given the significant length of diapause relative to the post-diapause lifespan, decisions to include or exclude diapause from age/lifespan measures have important consequences. The example of diapause thus illustrates that different methods of measuring the age of an animal can lead to drastically different ages for an individual animal or for maximum lifespan estimates.

Given the challenge of identifying a common starting point to determine age across species, the recently proposed “ground zero model of aging” is worth noting [53]. This model proposes that the resetting of epigenetic marks and other molecular features linked to aging that begin with the formation of germ cells is completed during early embryonic development, creating a “ground zero” or baseline for age-related molecular marks [53]. The model is based largely on findings by Gladyshev and colleagues that in mice, a 5-methyl-cytosine-based molecular clock records the lowest age for cells derived from the blastocyst stage, with cells from earlier and later stages recording higher methylation ages [53–55]. Molecular markers associated with aging other than cytosine methylation are also reset during early embryonic development; however, for most markers, extensive, time-resolved data similar to that used for cytosine methylation analysis are not available. Unfortunately, it is unknown if the “ground zero model of aging” applies to species other than mice. The most commonly used invertebrate aging models, *D. melanogaster* and *C. elegans* have no or very limited cytosine methylation [56, 57]. However, cytosine methylation is found in many invertebrate animals, but methylation clocks have not been tested extensively in these species (but see [58–60]). If a ground zero model—for cytosine methylation or another molecular marker—does apply to species other than mammals, then the “ground zero” time point might serve as the common starting point to determine age and allow for the comparison of equivalent age data between species.

For comparative aging studies across diverse animal species, the current approaches to measuring age and lifespan represent a significant problem. For these studies, age/lifespan measures must be compatible with each other to allow for meaningful comparisons. Given the complexities of animal life, solving this problem likely will require the development of adjustments to age measures recorded by currently used methods to bring them in alignment with each other. If a “ground zero” time point for aging markers exists in species across the tree of life [53], this time point could be used across species to measure aging in a way that facilitates comparative studies. However, additional complexities likely exist in species that undergo metamorphosis or experience diapause, requiring the development of new approaches to facilitate comparative studies of aging.

## Comparing lifespans across diverse species to identify equivalent life stages

A secondary challenge facing researchers interested in comparative studies of aging across evolutionary distant animal groups is how to identify appropriate time points for comparisons when the lifespans of animals can differ by two or more orders of magnitude, ranging from days to hundreds of years [61]. If we want to compare young to old animals, what then is the appropriate age of animals that should be collected from the various species? Assuming the first issue is resolved, and compatible age information is attainable, there is still a significant need to be able to use that information to define the time points that are appropriate to compare various physiological, molecular, and genomic phenotypes across diverse species.

**Fig. 2:**
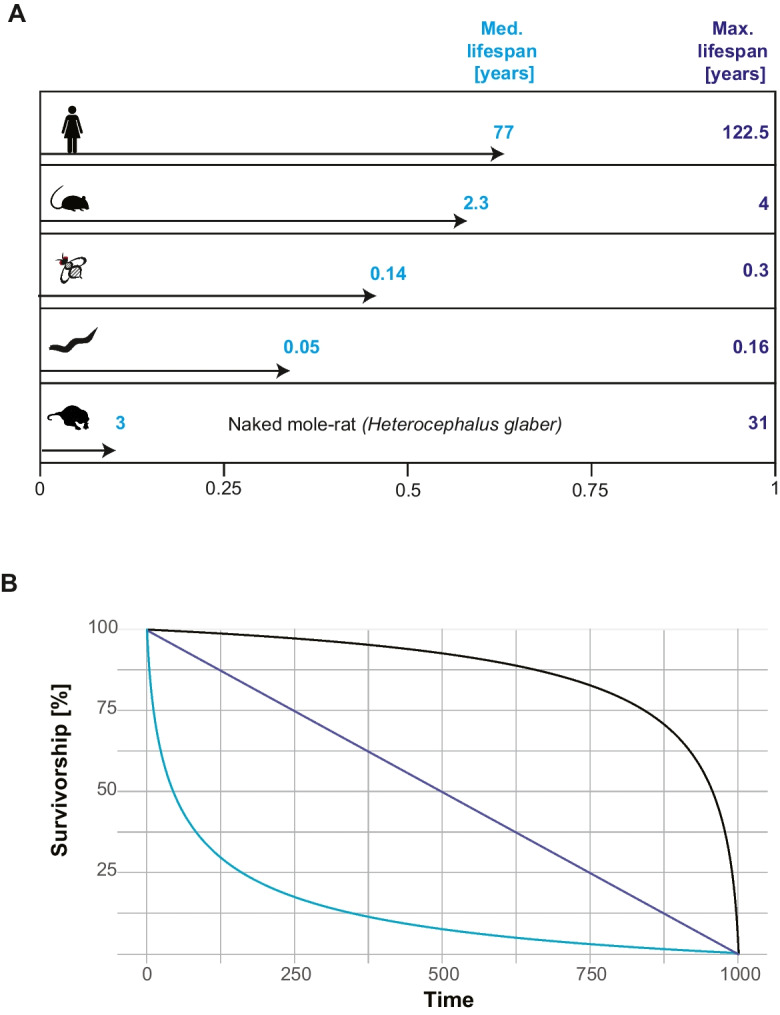
Approaches to identifying animals of comparable ages between species. **A** Relative age. Relative age is calculated by dividing an individual’s age by the maximum lifespan reported for the species. Thus, ages for any species are scaled to the range of 0–1. Above, this is illustrated for humans, mice, *Drosophila*, *C. elegans*, and the naked mole rat. The arrow indicates the median lifespan reported for these species, which highlights one of the problems of this approach. Depending on how much the maximum reported lifespan deviates from the median lifespan, it can be much harder or easier to collect animals in the upper ranges of relative age. **B** Percent survivorship. Percent survivorship is recorded starting with 100% of all animals being alive at the 0-time point and 0% of animals being alive at the maximum lifespan (1000) for the animal (time in arbitrary units on the X-axis; % survivorship on the Y-axis). Three idealized survivorship curves are shown. In black is a type I survivorship curve, where most individuals survive until fairly close to the maximum lifespan when the rate of death increases sharply and many animals die in a short period of time. In purple is a type II survivorship curve, where animals die at a more or less constant rate throughout their lifespan. Animal populations with a type III survivorship curve show high levels of deaths early in life, but after this period show much slower, relatively constant rates of deaths. While % survivorship can be used to compare species with a variety of survivorship curves, this diagram illustrates that for any given % survivorship, the animals might be in very different fractions of their total lifespan

Several approaches to this problem have been proposed in the literature, and one such measure used in comparative studies is “relative age” [62]. Relative age is defined as the actual age of the individual divided by the maximum lifespan reported for the species (RA = age/max lifespan). Relative age thus ranges from 0 to 1, independent of the species’ lifespan range (Fig. 2A). Relative age can be calculated for any individual of any species, as long as the individual’s age and a maximum lifespan for its species are available. Given that the maximum reported lifespans of many species can be found in the literature or in databases such as anAge [61, 63], relative ages are relatively easy to implement for comparative studies. A different means of calculating relative age uses transformations (such as z-transformation) to generate a units-free relativized age estimate to ensure that comparisons within and across species compare relatively older and younger (e.g. [64]).

A second approach to identifying comparable time points for studies of aging involving diverse species relies on estimating survival from life tables or mark-recapture studies [65]. Life tables record the % of individuals surviving from a cohort as a function of age [66]. At the 0 time point, 100% of all individuals are alive, and the percentage of individuals alive decreases over time until 0% are alive (Fig. 2B). With these data, it is then possible to identify specific points at which *X*% of all individuals are alive, irrespective of the actual age measure (day, weeks, year, etc.). Like relative age, this % survivorship has the same numerical range for all species (0–100%) and thus provides a way to select comparable time points for studies of aging involving species with different lifespans.

While both relative age and % survivorship provide a solution to how to identify comparable time points between species, there are challenges with both measures to be solved by individuals trying to implement these approaches. Both approaches rely on data being available for the species of interest, either life tables or maximum lifespan. However, it is often unclear if the data being used are compatible with the particular study on comparative aging in question (e.g., Lorenzini and colleagues use 90 years as the maximum human lifespan instead of the reported 122 years to adjust for the fact that the sample size from humans is much bigger than for the other species included in their study [67]). From studies both in the laboratory and in natural populations, it is clear that life tables and maximum lifespan are highly variable within a species and can change significantly depending on environmental factors such as temperature, food availability, or population density [68–71]. In addition, sex and genotype also strongly impact life tables and maximum lifespan [68–71]. For example, a study of approximately 200 genetically distinct *Drosophila melanogaster* strains found that the lifespan differed significantly between the strains, males and females, and between the three rearing temperatures used [72]. In addition, the rearing temperature strongly impacted the variability in lifespan, with higher temperatures leading to less variability than lower temperatures, and the sex differences in aging being dependent on both genotype and rearing temperature [72]. Similar temperature effects on estimated lifespan are observed in herbivorous marine fish across latitudinal gradients, where slower growth rates, larger body sizes, and greater estimated maximum lifespan are observed at lower temperatures [73]. These effects are observed also in garter snakes [74]. Furthermore, captivity often affects life tables and the maximum lifespan as social and environmental conditions are not present that lead to natural or wild population aging dynamics [75]. These studies illustrate the plasticity inherent in lifespan and life-table measures and suggest that likely, there is a large degree of currently undocumented variation in these measures for many species. This variability or plasticity in lifespan and life table measures raises the question of which life table or maximum lifespan should be used to calculate the % survivorship or relative age.

## Life history diversity within species poses additional challenges

In addition to the known environmental and genetic plasticity in lifespan and life-table measures, some species represent particular challenges, both for calculating relative age and for % survivorship. One example is the social insects, such as honey bees, *Apis mellifera*, that have different types or castes of individuals that have unique life histories and maximum lifespans [76]. In honeybees, male drones live 3–5 weeks. There are two female castes, queens and workers, which are full sisters and 75% genetically similar but differ significantly in lifespan. Queens live typically 2–3 years, and up to 8 years, while the worker bees’ lifespan depends on the season; they live 2–5 weeks in the summer, 4–9 weeks in the spring and fall, and 5–7 months in the winter. Because the majority of animals in a bee colony are workers, honeybee life tables tend to focus on them, and life tables for the other castes are rare. While there are life tables for males [77], we were unable to find life tables for queens with the exception of a small study of 15 queens over two years [78]. The extreme difference in lifespan between queen, worker, and drone bees illustrates the problem of using relative age with species that have large lifespan differences between different groups of individuals (sex, caste, etc.). If we use the maximum reported lifespan for all bees, that would be 8 years. However, with this maximum lifespan, drones only reach a relative age of 0.01, while workers reach 0.01–0.07, depending on which cohort they belong to (spring/fall, summer, or winter). Similarly, using the widely available life tables from worker bees for the other two castes would lead to problems, especially for the queen bees. Interestingly, a similar lifespan difference between the breeding animals and non-breeding workers is also documented for the naked mole-rat, *Heterocephalus glaber* (lifespan of up to 17 years for breeders and 2–3 years for workers), an exceptionally long-lived rodent (anAge database). These examples thus illustrate the challenges faced by researchers studying species groups with life histories that differ from the species that these tools were originally developed for.

Social insects, however, are not the only species of interest that present challenges when attempting to implement age comparisons with relative age and % survivorship measures. Any species where subsets of the population have significantly different life histories will pose the same challenge. Examples are species such as some fish, frogs, or spiders that have “sneaker” males. These males typically reach sexual maturity at an earlier time than the majority of males, and instead of participating in the usual competition for females that favors large males, their reproductive strategy is to circumvent this process and “sneak” matings, while the alpha male is unaware [79]. While extensive data are not always available, it is thought that sneaker males have a shorter lifespan and distinct survivorship curves from the other males [80–82]. In Atlantic salmon, males present two reproductive strategies, with one precocial tactic similar to “sneaker” males that reach sexual maturity earlier and reproduce in the river before migrating to sea to complete the anadromous life cycle [83]. Pacific salmon, including sockeye (*Oncorhynchus nerka*), coho (*Oncorhynchus kisutch*), and chinook (*Oncorhynchus tshawytscha*), exhibit alternative reproductive strategies where some males will return early (the same year they smolted) from the ocean (“jacks”) and successfully sneak-spawn despite their smaller size [84, 85]. Further complicating maximum lifespan calculations is the fact that most salmonid species undergo rapid senescence and die shortly after spawning. In some species, there are reports that some sneaker males might change reproductive strategies later in life and start to participate in the standard male–male competition for females, creating more complex survivorship curves and maximum lifespan. Therefore, in species with sneaker males, there are potentially three different types of males present that have distinct survivorship curves and maximum lifespan, and it might not be easy to assign individuals to the specific type or place them appropriately within a proper curve. Calculating both relative age or selecting individuals for studying these species using % survivorship is fraught with challenges, and the best approach is not clear.

An additional case that poses unique challenges in comparative studies is the occurrence of distinct morphological subtypes or morphs in some classes of animals, which present with morph-specific traits including lifespan. Many animals, including a range of insects, have distinct morphs in the fall that then overwinter. Some of the most well-known of these types of insects are likely the monarch butterfly, which has a fall morph that migrates to its winter habitat and overwinters [86], and the locust, which can be either solitary or migratory and swarm-forming [87]. The seasonal morphs in these species can differ in their maximum lifespan and their survivorship curves, but more often, extensive datasets for the various morphs are lacking. Cicadas provide another interesting example, as there are annual morphs and there are morphs that spend either 13 or 17 years as nymphs underground [13]. Thus, for these types of species, it is difficult to determine how to use relative age or percent survivorship to identify individuals of comparable ages.

Together, these examples demonstrate that while relative age and percent survivorship are established methods used in comparative studies of aging, implementing their use in studies across evolutionarily distant species groups is not always straightforward. While all species will show some variability in maximum lifespan and their survivorship curves in response to environmental factors, sex, and genotype, in some species, these differences are extreme due to the existence of distinct subpopulations with divergent life histories. Including these types of species in comparative studies of aging might require new approaches or modifications to the standard relative age or percent survivorship.

## Challenges and opportunities

In an ideal situation, researchers would have access to reliable lifespan tables, survivorship graphs, and maximum lifespan measurements derived from a large sample size available for the specific population, morph, genotype, or sex under study collected under the same environmental conditions. They would be able to assess phenotypes of interest in individuals from across the entire lifespan to understand how these phenotypes change with age. Comparison between species could then be set up by comparing trajectories and patterns rather than being based on individual data points that might or might not be equivalent.

Unfortunately, for many species, the data ideally required are not available, and instead, research designs are based on whatever data are available. Due to funding constraints, comparative studies of aging typically include few time points per species (often less than 5), meaning that the choice of time points for comparisons has important implications for the outcome of these studies. Given the challenges outlined here, it might be worth considering using multiple approaches to compare age-associated phenotypes between species. This strategy would mean, for example, identifying equivalent percentile-based time points based on only the adult lifespan and also based on the entire lifespan from fertilization and contrasting the results. It could also include considering both relative age as well as percent survivorship in comparative studies of aging. While the use of multiple approaches can be costly, it is likely to lead to new insights. To explore these ideas and develop additional approaches, existing datasets could be re-analyzed to initiate further dialog in the comparative aging field.
